# Early maturation and liver necrosis in the fingerling stage of *Oreochromis mossambicus* due to BPA can cause an ecological imbalance†

**DOI:** 10.1039/c7ra11432j

**Published:** 2018-04-05

**Authors:** Abhijit Manna, Chinnaiah Amutha

**Affiliations:** Department of Animal Behaviour and Physiology, School of Biological Sciences, Madurai Kamaraj University Madurai-625 021 India amuthaji@gmail.com +91-452-2459139 +91-452-2458246

## Abstract

We investigated the effect of Bisphenol-A (BPA) on the fingerlings of *Oreochromis mossambicus* collected from industrial waste. Fluorescence quenching assay using Rhodamine-B and mass detection assay using ESI-MS revealed that BPA was predominantly present in plastic industry effluent, where the fingerlings' ovaries exhibited early maturation. The histopathology of those fingerlings revealed a similar result. Both quantitative and qualitative data obtained by ELISA and FPLC showed elevated levels of vitellogenin in the fingerling stages after prolonged exposure to BPA present in the contaminated water. Our qRT-PCR data showed a subsequent increased expression of vitellogenin in those fingerlings obtained from contaminated effluent. FACS analysis suggested that BPA generated a significant amount of ROS in the livers of those fingerlings, leading to necrosis in hepatocytes.

## Abstract 2

We investigated the effect of Bisphenol-A (BPA) on the fingerlings of *Oreochromis mossambicus* collected from industrial waste.

## Graphical abstract

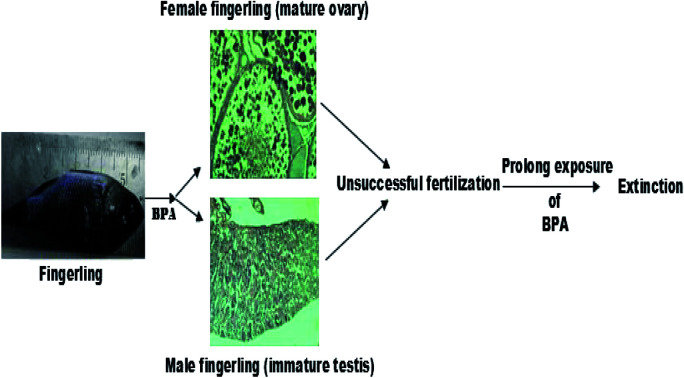

## Introduction

The role of xenobiotic compounds in water pollution and their harmful effects on living organisms is well known. However, there is no such in-depth investigation and remedial measures available against these xenobiotic compounds. Industrial waste is the primary source of endocrine disrupting chemicals (EDCs). The endocrine system is one of the most intricate and essential systems that regulates different essential hormones used in various cellular functions.^1^ Endocrine disrupting chemicals can alter the endocrine system by affecting these hormones. Some EDCs can resemble key hormones^2^ and as a consequence, the hormonal regulation can be altered.^3^ Exposure to EDCs has been associated with numerous adverse reproductive defects,^4,5^ such as reduction in female fecundity as well as male sperm motility, longer time to conception, and massive miscarriage rates.^6,7^ Aksglaede *et al.*, 2009 and Roy *et al.*, 2009 reported that, the age at menarche advanced slowly in females' mostly in developing countries and there might be a role of EDCs.^8,9^ EDCs have been found to delay the sexual development of fish.^10–13^

Bisphenol-A [(4,4′-propane-2,2-diyl)diphenol] is a phenolic compound containing two benzene rings along with two OH substituents, which can fit perfectly on the estrogen receptor. Therefore, it behaves like an environmental xenoestrogen. Bisphenol-A is usually present in low-quality plastics and leaches when these plastics are in contact with water.^14,15^ Plastic industry discharge units are considerable sources of BPA, causing environmental pollution. Different kinetic parameters have revealed that BPA competes with estrogen for its receptor even at a micromolar level and causes grave consequences.^16^ BPA is a causative agent of breast cancer^17^ and also contains potential antagonistic androgenic activity.^18–20^

Fish are considered as a convenient animal model to study the effect of xenobiotics in the aquatic ecosystem. There are various reports stating that *Oreochromis mossambicus* (Mozambique tilapia) and *Oreochromis niloticus* (Nile tilapia) are the predominant model freshwater fish. Apart from their fast growth rate, water quality, heavy metal toxicity, and oxidative stress in the aquatic environment can be easily detected from their physiological and genetic changes.^21,22^ Juvenile *Oreochromis mossambicus* are used as an animal model for EDC assay because it is easy to measure the levels of vitellogenin (Vtg) mRNA in the early developmental stages where the expression of vitellogenin is low.^21,23^ Other than consumption through diet, fish can absorb BPA through their gills, where mostly BPA gets deposited, but metabolize. Even though liver has its own detoxification limit, BPA deposition takes place if the concentration of BPA in the liver exceeds its detoxification limit. Moreover, defects in both BPA metabolism and its deposition may trigger estrogenic modulation, including excessive secretion of vitellogenin (egg yolk precursor proteins) in females and a minute secretion quantity in males.^24^ Vitellogenin (Vtg) is a wide-range molecular weight (250–600 kDa) calcium-binding egg yolk protein, which is essential for oocyte maturation.^25^ Vtg is usually produced due to estrogenic stimulation in the liver and then released into the blood. BPA contamination in water leads to oxidative stress, destruction of macrophages (dysregulation of the immune system) and early maturation of ovaries in fish. We investigated the endocrine disrupting effect of BPA on *Oreochromis mossambicus* collected from BPA contaminated water.

## Materials and methods

### Detection of BPA in different effluent samples

Water samples polluted with the effluent of industrial wastes from industries such as electroplating, tanning, rubber, paper, steel, dye, fertilizer, textile and plastic were collected. For each differently polluted effluent, 20 mL of sample was mixed with an equal volume of ethyl acetate (analytical grade), followed by vigorous shaking. After extraction, the organic part of all the samples was collected. Any remaining aqueous part, which entered in to the organic part, was removed by adding anhydrous sodium sulfate.^26^ The fluorescence of Rhodamine-B^27^ is usually quenched in the presence of BPA. After extraction, the organic part of individual industrial effluent was mixed with an equal volume of Rhodamine-B (10 nM) and the emission spectra of all extracted samples were recorded at 625 nm along with that of standard Rhodamine-B.^28^ The degree of quenching is directly proportional to the concentration of BPA present in that specific effluent sample.^20^ After ethyl acetate extraction, all samples were kept at room temperature for complete evaporation, followed by mixing with methanol (MS grade) and then, the samples were analysed using ESI-MS. The ESI-MS data were compared against a standard ESI-MS peak of BPA to confirm its presence.^20^ Generally, in ESI-MS, the molecule of interest ionizes and thus gains either positive or negative charge and then, the ions will travel through the mass analyzer, where they will be detected on the basis of mass/charge ratio (*m*/*z*). Relative abundance is displayed as a result according to *m*/*z* ratio.^29^

### Effect of BPA on the fingerlings of *Oreochromis mossambicus* (Mozambique tilapia) and detection of differential changes

Fingerlings of *Oreochromis mossambicus* were collected from natural water bodies, such as ponds, contaminated with different industrial effluents. Usually, fishermen released the spawns of *Oreochromis mossambicus* in these water bodies for farming in a natural environment and after two months, they grow into fingerlings; alternatively, the fishermen directly released the fingerlings in the waterbodies. In most cases, specific industrial effluents either directly contaminate these water bodies or after absorption in the soil, it will contaminate the nearby water bodies, mostly during the rainy season. As a result, aquatic organisms such as fish are severely affected. According to the fishermen's information, for this study, all fingerlings were collected from different contaminated water bodies after one month of exposure.

Ten fingerlings (equal number of male and female) of *Oreochromis mossambicus* were collected separately from water bodies (natural growth environment) contaminated by different industrial effluents. Similarly, equal numbers of male and female fingerlings were collected from non-contaminated water bodies (natural growth environment) to serve as controls. All fingerlings were dissected using sterile dissection apparatus. In addition, as a positive control in semi-natural conditions, healthy fingerlings collected from a fish farm were grown in a cement tank with BPA treatment (200 ppm concentration) for one month. Ovaries and testes were carefully observed for the presence of maturation or abnormalities. Liver, testis and ovary samples of different sets of fingerlings were collected from contaminated and non-contaminated water washed in 1× PBS (pH 7.4), followed by storage in 25% formalin and were subsequently sent for histopathological slides' preparation. Similarly, liver, testis and ovary samples of positive control fingerlings were also sent for histopathological analysis. During histological slides' preparation, the tissue was first fixed in 10% formalin solution, followed by dehydration using alcohol. Subsequently, it was embedded in wax and sectioned using a microtome. Finally, sectioned tissues were mounted on clean slides and stained by applying hematoxylin-eosin.^30^ Histopathology slides were observed carefully under a light microscope and compared with the control (fingerling without exposure to BPA contaminated water) and the positive control (fingerling with exposure to BPA in semi-natural conditions) to identify the deformities caused due to BPA contamination. Vitellogenin is a biomarker of ovary maturation secreted in the adult stage of fish; however, the endocrine disrupting effect of xenoestrogens such as BPA may also cause its secretion. Due to pollution by BPA, vitellogenin expression increased in the early fingerling stage, indicating endocrine disruption. The increased level of vitellogenin during the early fingerling stage can be used as a biomarker to study the endocrine disrupting effect caused due to EDCs like BPA. Simultaneously, xenobiotic compounds can enhance the secretion of aromatase. Liver samples collected from different fingerlings were crushed gently in PBS (pH 7.4) using sterile mortar and pestle, followed by centrifugation at 5000*g* for 10 minutes. Then, 65% fraction from ammonium sulfate precipitation was run in DEAE-cellulose^31^*via* fast protein liquid chromatography. The peak appearing in the NaCl gradient line was collected separately and confirmed using a vitellogenin specific ELISA kit [CUSABIO Grouper Vitellogenin (VTG) ELISA Kit-CSB-EI4116Fh].^2,32^ A plasma sample was used in a similar manner for the detection of vitellogenin secretion. Aromatase is secreted from the brain in the presence of xenobiotic contaminants. Following the same procedure as the liver sample, the brains were collected from the same sets of fingerlings, crushed into the same buffer, and centrifuged at 5000*g* for 10 minutes. The resultant supernatant was used for ELISA using the aromatase specific ELISA kit [CUSABIO Cytochrome P450 19A1 (CYP 19A1)-CSB-EL006394HU].^20^ The concentration of vitellogenin secretion from liver and plasma along with aromatase secretion from the brains were compared with control fingerlings and statistically analyzed using one-way ANOVA.

Expression of vitellogenin gene in both control and contaminated fingerlings was determined using qRT-PCR. Liver samples were stored in RNA later solution to halt RNase activity. Then, 100 mg of the liver sample was homogenized using a sterile micropestle, followed by the addition of 500 μL of TriZol and incubation at room temperature for 5 minutes. After incubation, 200 μL of chloroform was added and mixed vigorously, followed by incubation at room temperature for further 10 minutes. Subsequently, the sample was centrifuged at 12 000 rpm for 15 minutes at 4 °C and then, the supernatant was transferred into a fresh microfuge tube before the addition of 500 μL of isopropanol. The sample was incubated at room temperature for 10 minutes for precipitation, followed by centrifugation at 13 000 rpm for 10 minutes at 4 °C; then, the pellet was washed with 150 μL of 75% ethanol. The dried pellet was resuspended in 50 μL of DEPC treated water and stored at −80 °C. DNase-treated RNA was used as a template for the synthesis of first strand cDNA. First strand cDNA was constructed using RevertAid H Minus First Strand cDNA synthesis kit (Thermo SCIENTIFIC-K1621) for all fingerlings and the differential vitellogenin gene expression was quantified using an ABI Prism 7000 qRT- PCR machine.^2^ Each 10 μL qRT-PCR reaction contained 5 μL of 2× SYBr Green Mix, 1 μL of cDNA, 2 μL of MilliQ, 1 μL of (10 pmol μL^−1^) forward and reverse vitellogenin gene (Vtg) specific primers (F-TCGAGCTGGGGTTAAAATC, R-TGGCAGTGGTTCAGGTC).^22^ The thermal cycle program was set to 94 °C (3 minutes), followed by 35 cycles of 94 °C (30 seconds), 55 °C for (30 seconds), and 72 °C (45 seconds).^23^ Each sample was evaluated in at least triplicate. β-actin was used as an internal control and the data was analyzed *via* ΔΔCT method.^33^ The vitellogenin gene expression fold change was statistically analyzed using one-way ANOVA.

### Reactive oxygen species generation in liver

Liver samples obtained from different sets of fingerlings were crushed in phosphate buffered saline (pH 7.4). The entire crushed sample was centrifuged for 10 minutes at 10 000 rpm at 4 °C. The supernatant was collected, following which 10 μM 2′,7′-dichlorofluorescin diacetate was added and then, the sample was subjected to FACS (fluorescence activated cell sorting) analysis to detect ROS (reactive oxygen species). In FACS, cells in a liquid stream pass through a laser beam in a single file and the interaction with light is measured as light scattering and fluorescence. ROS are generated in cells due to several reasons; toxic chemicals are one of the predominant inducers of ROS. The compound 2′,7′-dichlorofluorescin diacetate is a widely used ROS indicator; it gets oxidised, converted into fluorescent 2′,7′-dichlorofluorescin, and it will be determined in FACS.^34,35^

### Ethical statement

Fish were maintained in accordance with the guidelines of the American Fisheries Society (guidelines for the use of fish, 2014) and approved by the Institutional Ethical Committee of Madurai Kamaraj University [Internal Research and Review Board (IRB), Ethical Clearance (EC), Biosafety and Animal Welfare Committee].

## Results and discussion

### Detection of BPA in the effluents and its effect on *Oreochromis mossambicus*

**Fig. 1:**
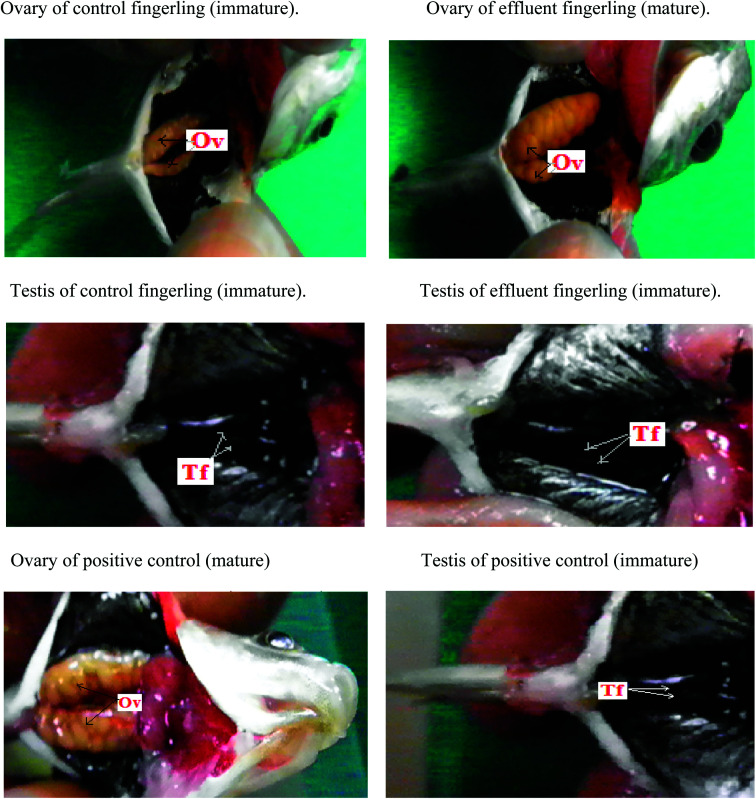
Dissection images clearly revealed that ovary (Ov) of control fingerling is immature but the ovary of the fingerling collected from plastic industry effluent showed early maturation like positive control because of the endocrine disrupting activity of BPA (estrogen mimicking role) but testis (Tf) of control along with fingerling caught from plastic industry effluent clearly manifested the immaturity because of the antagonistic role of BPA against androgen.

The maximum quenching of Rhodamine-B fluorescence was observed in the contaminated water sample collected from plastic industry effluent (Fig. Sp-1†) and it was almost confused with the fluorescence quenching that occurred in presence of 200 ppm concentration standard BPA.^28^ ESI-MS data of the plastic industry effluent sample revealed that the intensity of BPA peak at *m*/*z* 227 was similar to (Fig. Sp-2†) the standard 200 ppm BPA peak. All other industrial effluent samples showed the presence of BPA, but the highest amount of BPA was found in the effluent from the plastic industry. All fingerlings (ten fingerlings (equal number of male and female) each from every site) collected from each of the various contaminated sites were analyzed. Experiments were performed in triplicate (figure not given). High level of EDC was found in the fingerlings that were contaminated and collected from plastic industry effluent. Hence, this study was focused on the effect of the plastic industry effluent on fingerlings. After dissection, similar to the positive control (Fig. 1), fully matured eggs were observed in the ovaries of female fingerlings collected from water polluted with the plastic industry effluent, but the ovaries of control fingerlings manifested no abnormal (immature) conditions and remained immature, which is expected at this stage. Moreover, both control and effluent testes were normal. Histopathology slides under light microscopy revealed that ovaries of the fingerlings obtained from the most polluted water appeared like mature ovaries, similar to the positive control (Fig. Sp-6†). However, in the controls, the ovaries remained normal (immature) (Fig. Sp-3†).

Due to the acidic nature of BPA, hepatocytes were lysed in both male and female fingerlings collected from plastic industry effluents; however, hepatocytes were normal in the livers of control fingerlings (Fig. Sp-4†). The histopathology of the testes from effluent and control fingerlings exhibited normal (immature) conditions (Fig. Sp-5†). During histopathological analysis, liver, ovary and testis samples from all ten fingerlings collected from each effluent were used. Two peaks (P-1, P-2) appeared in FPLC (Fig. Sp-7†) after running the 65% ammonium sulfate saturated fraction of the liver in a DEAE prepacked column. The fractions corresponding to those two peaks were used for ELISA using the vitellogenin specific ELISA kit and it was clear that peak one (P-1) appeared due to vitellogenin. In addition, the liver fraction of the control fingerlings did not show any distinguishing peak; this may be due to low production of vitellogenin. Separately, liver and plasma samples were used to detect the quantity of vitellogenin production using ELISA. It was clear that female fingerlings collected from plastic industry effluents manifested early maturation due to endocrine disruption.

**Fig. 2:**
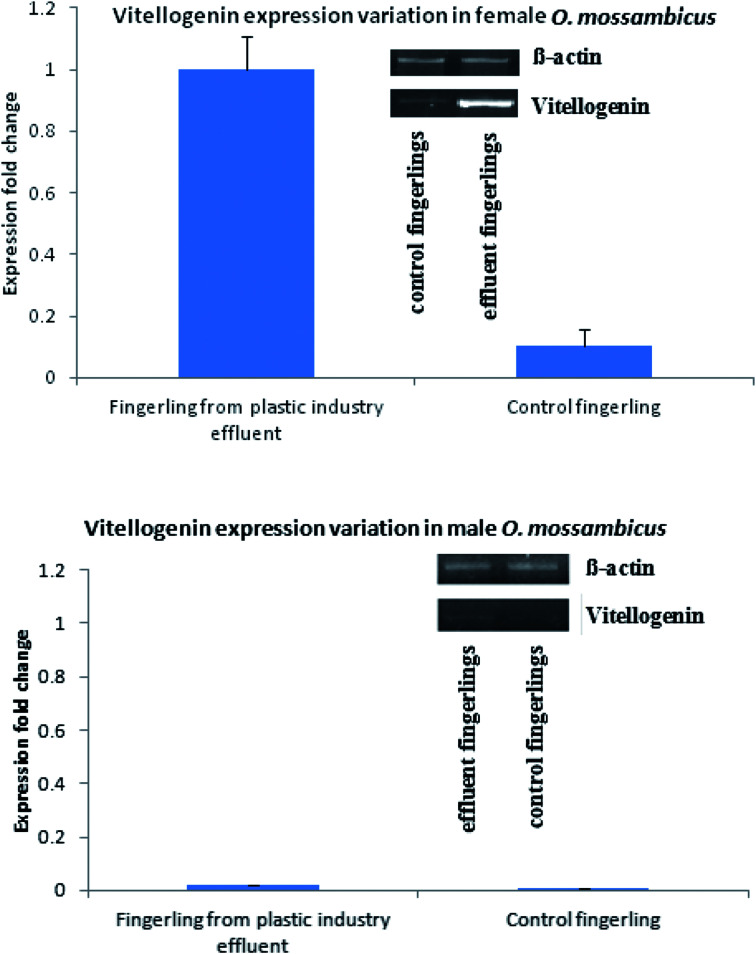
Quantitative real time PCR showed that female fingerling collected from plastic industry effluent showed up-regulation of vitellogenin with respect to β-actin gene; in case of male, although vitellogenin is down-regulated, expression has been increased with respect to control.

Similarly, production and quantity of aromatase was very low in control fingerlings when compared to fingerlings collected from the plastic industry effluents-contaminated water. Statistically significant differences were observed in the production of both vitellogenin and aromatase (*P* < 0.001). This indicates that both vitellogenin and aromatase production was significantly higher in fingerlings exposed to the plastic industry effluent (Fig. Sp-8–Sp-10†). This is due to the endocrine disrupting effect of BPA present in ample quantity in the plastic industry effluent. Quantitative real-time PCR (Fig. 2) also revealed that the vitellogenin gene expression in female fingerlings captured from the same contaminated water was up-regulated as compared to β-actin gene expression, while the converse was true in control fingerlings (*P* < 0.001). In male fingerlings, vitellogenin gene expression was not up-regulated, but fingerlings exposed to plastic industry effluent showed higher expression of vitellogenin than the controls. During the quantitative real-time PCR experiment, RNA was extracted from ten fingerlings (equal number of male and female) and this experiment was performed in triplicate.

### Generation of reactive oxygen species in liver

**Fig. 3:**
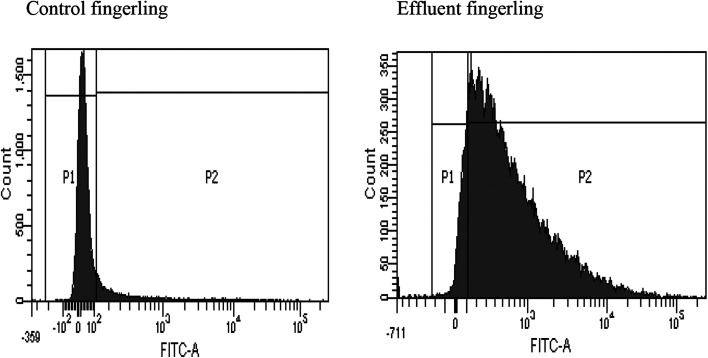
ROS generation detection in the liver of effluent fingerlings using FACS.

FACS analysis showed that the liver samples of effluent exposed fingerlings contained 89% of ROS (reactive oxygen species), but in the control sample, no significant ROS generation was observed. This experiment was conducted to detect the cause for necrosis in the livers of effluent contaminated fingerlings. Apart from its phenolic and acidic nature, BPA can form a significant amount of ROS inside the liver.^36^ This could have triggered the necrosis of hepatocytes as observed in Fig. 3. This study clearly demonstrated the estrogenic role of BPA causing early maturation in the female fingerlings. Moreover, due to the antagonistic role of BPA against androgen, the testes remained immature and severe necrotic effect on hepatocytes was also observed due to drastic ROS generation.^37^

## Conclusion

BPA is a predominant endocrine disrupting chemical with estrogenic activity, favoured by its structural similarity to estrogen and higher binding affinity with the estrogen receptor. BPA is found in both domestic and industrial sites. In the modern era, most of the plastics industries are using low quality epoxy resins. This consequently generates adequate quantity of BPA when released in water bodies even for a moderate period of time. ESI-MS and Rhodamine-B fluorescence quenching assay clearly showed that the quantity of BPA is significantly higher in the plastic industry effluent than in other effluents. Histopathology analysis of the female fingerlings of *Oreochromis mossambicus* exhibited early mature ovaries with fully matured eggs although male fingerlings were immature. Also, the liver histopathology showed necrotic hepatocytes along with ample quantity of ROS due to BPA in the liver. Subsequently, it appears that the consumption risk of BPA has been increased. BPA is silent killer as it can seriously affect life forms by causing ecological destabilisation.

We suggest that BPA contamination can cause prematuration in the ovaries of *Oreochromis mossambicus* when exposed for longer periods due to estrogenic activity. In addition, the antagonistic role of BPA against androgen caused atrophied testes in the male fingerlings. This can distort the reproductive scenario of *Oreochromis mossambicus* due to an imbalance in the sex ratio. Thus it poses a threat of endangering the species because only female maturation cannot fulfil reproductive success. Considering this as a model species of toxicological analysis, this study suggests that BPA can produce a similar effect on other species. As a result, ecological destabilization can occur. We suggest that necessary measures should be taken to prevent the ecological imbalance caused by endocrine disrupting substances such as BPA.

## Conflicts of interest

There is no conflict of interest.

## Supplementary Material

RA-008-C7RA11432J-s001

## References

[cit1] Mnif W., Hassine A. I. H., Bouaziz A., Bartegi A., Thomas O., Roig B. (2011). Int. J. Environ. Res. Public Health.

[cit2] Manna A., Geetha S., Tamilzhalagan S., Amutha C. (2016). RSC Adv..

[cit3] National Insitute of Environmental Health Science , Endocrine. Disruptors, 2010

[cit4] Gray L. E., Ostby Jr J., Sigmon R., Ferrell J., Rehnberg G., Linder R., Cooper R., Goldman J., Laskey J. (1988). Reprod. Toxicol..

[cit5] Gray L. E., Ostby Jr J., Ferrell J., Sigmon R., Cooper R., Linder R., Rehnberg G., Goldman J., Laskey J. (1989). Prog. Clin. Biol. Res..

[cit6] Singleton D. W., Khan S. A. (2003). Front. Biosci..

[cit7] Crain D. A., Janssen S. J., Edwards T. M., Heindel J., Ho S. M., Hunt P., Iguchi T., Juul A., McLachlan J. A., Schwartz J., Skakkebaek N., Soto A. M., Swan S., Walker C., Woodruff T. K., Woodruff T. J., Giudice L. C., Guillette L. J. (2008). Fertil. Steril..

[cit8] Aksglaede L., Sorensen K., Petersen J. H., Skakkebaek N. E., Juul A. (2009). Pediatrics.

[cit9] Roy J. R., Chakraborty S., Chakraborty T. R. (2009). Med. Sci. Monit..

[cit10] Den Hond E., Roels H. A., Hoppenbrouwers K., Nawrot T., Thijs L., Vandermeulen C., Winneke G., Vanderschueren D., Staessen J. A. (2002). Environ. Health Perspect..

[cit11] Denham M., Schell L. M., Deane G., Gallo M. V., Ravenscroft J., De Caprio A. P. (2005). Pediatrics.

[cit12] Vandenberg L. N., Maffini M. V., Sonnenschein C., Rubin B. S., Soto A. M. (2009). Endocr. Rev..

[cit13] Kendig E. L., Le H. H., Belcher S. M. (2010). Int. J. Toxicol..

[cit14] Saiyood S., Vangnaia S., Thiravetyan P., Inthorn D. (2010). J. Hazard. Mater..

[cit15] Rajasärkkä J., Pernica M., Kuta J., Lašňák J., Šimek Z., Bláha L. (2016). Water Res..

[cit16] Gould J. C., Leonard L. S., Maness S. C., Wagner B. L., Conner K., Zacharewski T., Safe S., McDonnell D. P., Gaido K. W. (1998). Mol. Cell. Endocrinol..

[cit17] Arboleda C., Cabana H., De Pril E., Peter Jones J., Jiménez G. A., Mejía A. I., Agathos S. N., Penninckx M. J. (2013). ISRN Biotechnol..

[cit18] Lee H. J., Chattopadhyay S., Gong E. Y., Ahn R. S., Lee K. (2003). Toxicol. Sci..

[cit19] Sun H., Xu L. C., Chen J. F., Song L., Wang X. R. (2006). Food Chem. Toxicol..

[cit20] Manna A., Amutha C. (2017). Environ. Sci.: Nano.

[cit21] Ayadi I., Monteiro S. M., Regaya I., Coimbra A., Fernandes F., Manuel Oliveira M., Peixoto F., Mnif W. (2015). RSC Adv..

[cit22] Sweidan A. H., El-Bendary N., Hegazy M. O., Hassanien E. A., Snaself V. (2015). Procedia Comput. Sci..

[cit23] Esterhuysea M. M., Venterb M., Veldhoenc N., Helbingc C. C., vanWyk J. H. (2009). J. Steroid Biochem. Mol. Biol..

[cit24] Kang J. H., Aasi D., Katayama Y. (2007). Crit. Rev. Toxicol..

[cit25] Sumpter J. P., Jobling S. (1995). Environ. Health Perspect..

[cit26] Staples S. A., Dorn P. B., Klecka G. M., O'Block S. T., Branson D. R., Harris L. N. (2000). Chemosphere.

[cit27] Gui-ping C., Ting C., Ya-feng Z. (2013). J. Fluoresc..

[cit28] Mechichi H. Z., Mechichi T., Dhouib A., Sayadi S., Martínez A. T. (2006). Enzyme Microb. Technol..

[cit29] Ho C. S., Lam C. W. K., Chan M. H. M., Cheung R. C. K., Law L. K., Lit L. C. W., Ng K. F., Suen M. W. M., Tai H. L. (2003). Clin. Biochem. Rev..

[cit30] Leica Biosystem, An introduction to specimen preparation

[cit31] Bartell S. E., Schoenfuss H. L. (2012). ISRN Toxicol..

[cit32] Livak K. J., Schmittgen T. D. (2001). Methods.

[cit33] Gomes A., Fernandes E., Lima J. L. (2005). J. Biochem. Biophys. Methods.

[cit34] Zeinab K. H., Mai A. E., Promy V., Sawsan A. O., Maha A. E., Maha H. D., Ebtisam M. A. O. (2012). Oxid. Med. Cell. Longevity.

[cit35] Sharpless T., Traganos F., Darzynkiewicz Z., Melamed M. R. (1975). Acta Cytol..

[cit36] Jaeschke H. (2011). J. Gastroenterol. Hepatol..

[cit37] Jaeschke H., Ramachandran A. (2011). J. Hepatol..

