# Supplementary material for: Early maturation and liver necrosis in the fingerling stage of Oreochromis mossambicus due to BPA can cause an ecological imbalance
Source: RSC Adv. 2018 Apr 5;8(23):12894–9. doi: 10.1039/c7ra11432j (PMC9079619; doi:10.1039/c7ra11432j)
Supplement: RA-008-C7RA11432J-s001 [file RA-008-C7RA11432J-s001.pdf]

Electronic Supplementary Material (ESI) for RSC Advances.
This journal is © The Royal Society of Chemistry 2018

Supplemantary Figure.

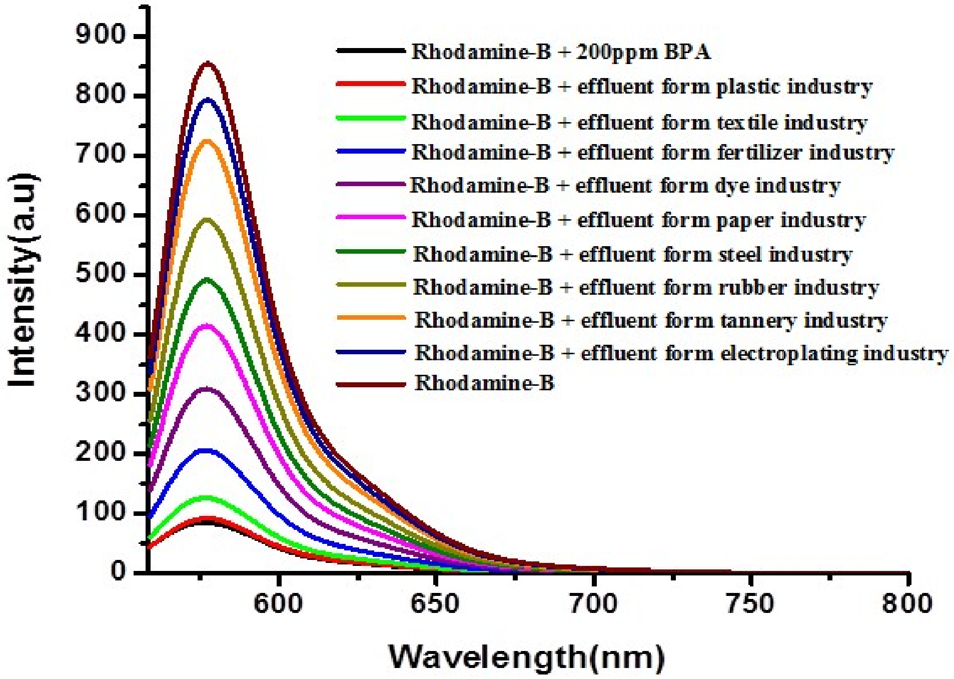

Figure: Sp-1) Detection of BPA in different effluent samples using Rhodamine-B.

(Specific peak appeared due to standard BPA).

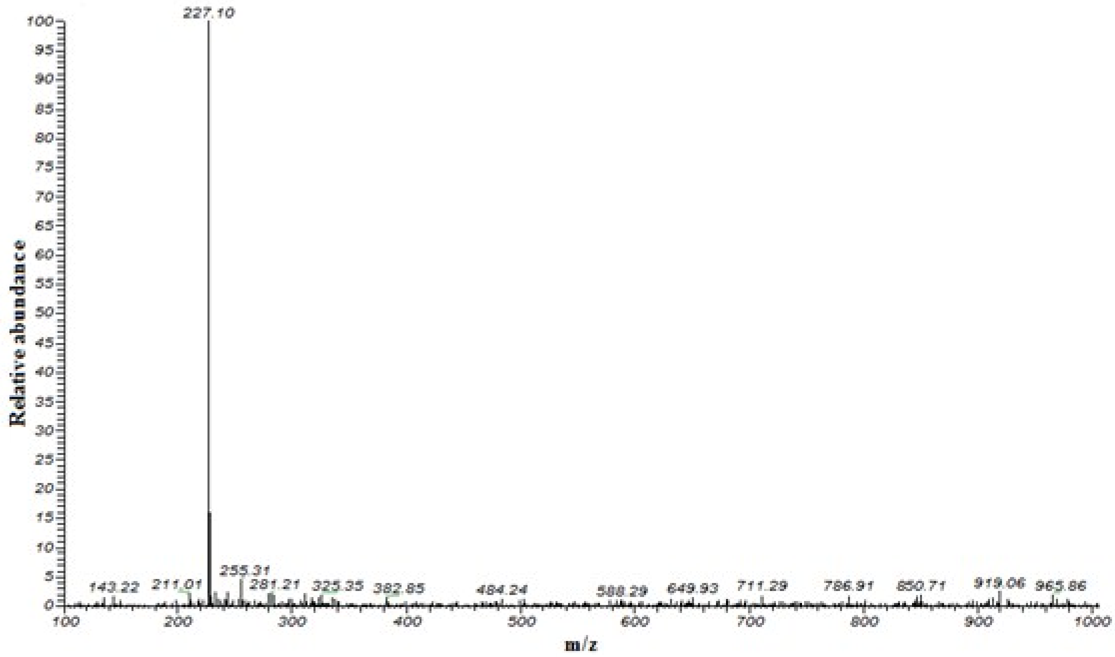

(Specific BPA peak appeared in plastic industry effluent sample).

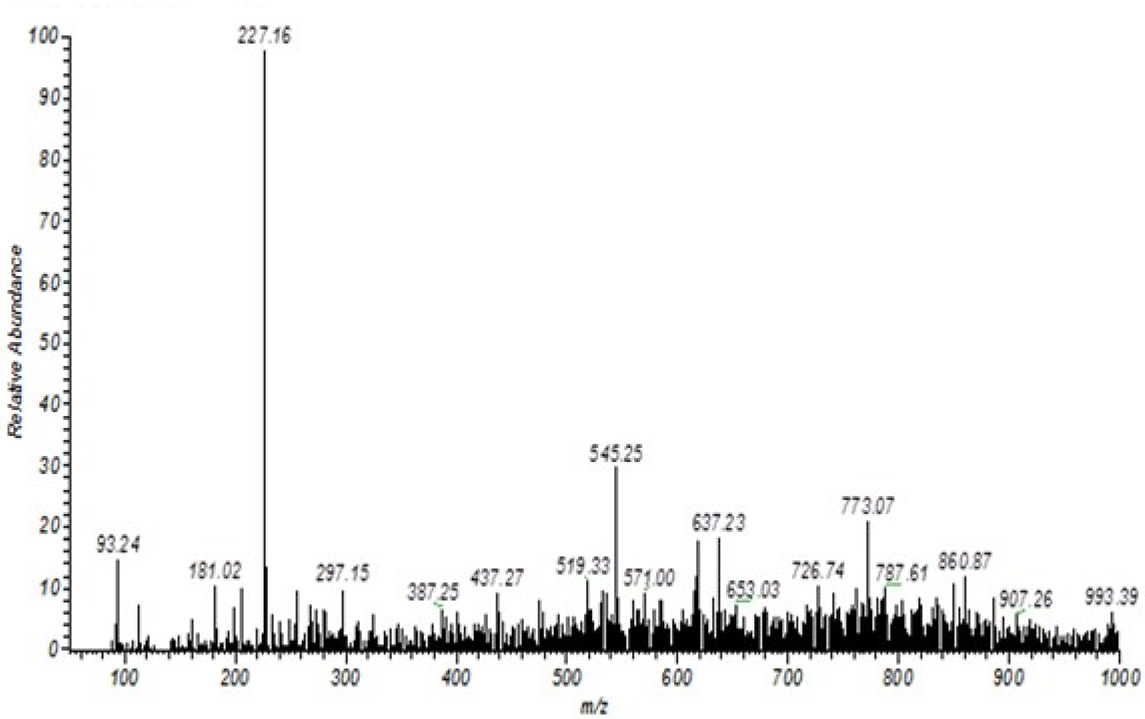

Figure: Sp-2) Conformation of BPA in plastic industry effluent using ESI-MS.

a) (Normal ovary of control fingerling) b) (Mature ovary of effluent fingerling)

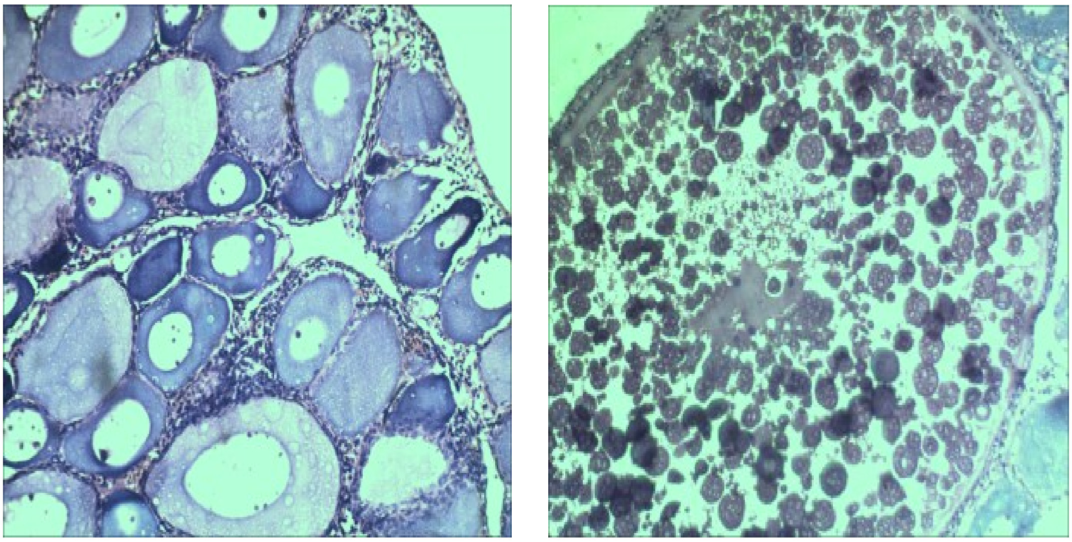

Figure: Sp-3) Histopathology of (a) control and (b) effluent fingerling's ovary.

a) (Normal liver of control fingerling) b) (Necrotic liver of effluent fingerling)

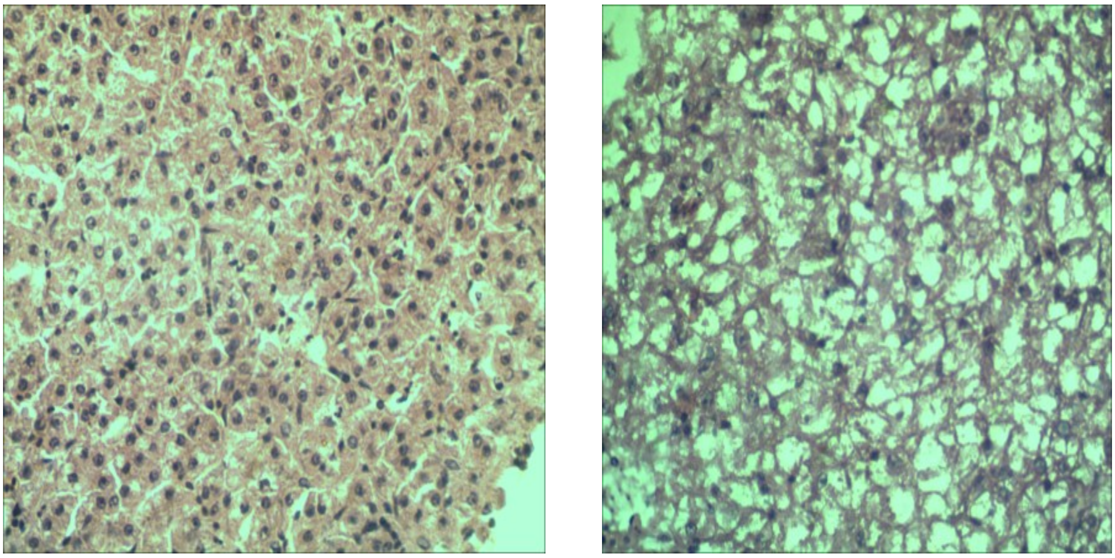

Figure: Sp-4) Histopathology of (a) control and (b) effluent fingerling's liver.

a) (Normal testis of control fingerling) b) (Normal testis of effluent fingerling)

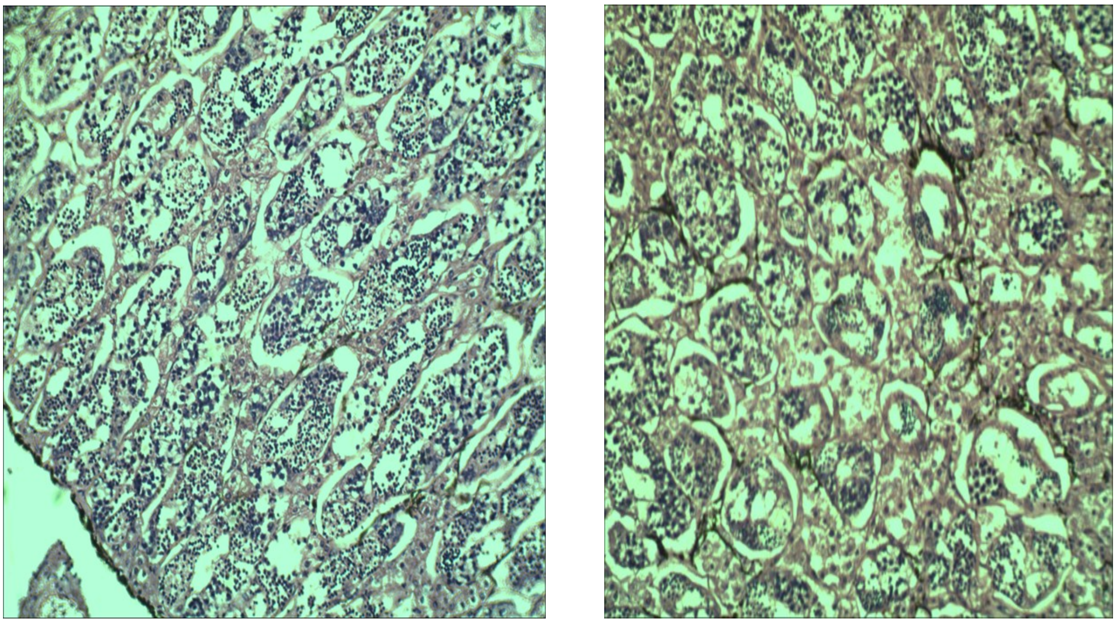

Figure:Sp-5) Histopathology of (a) control and (b) effluent fingerling's testis.

a) Complete mature ovary of positive control

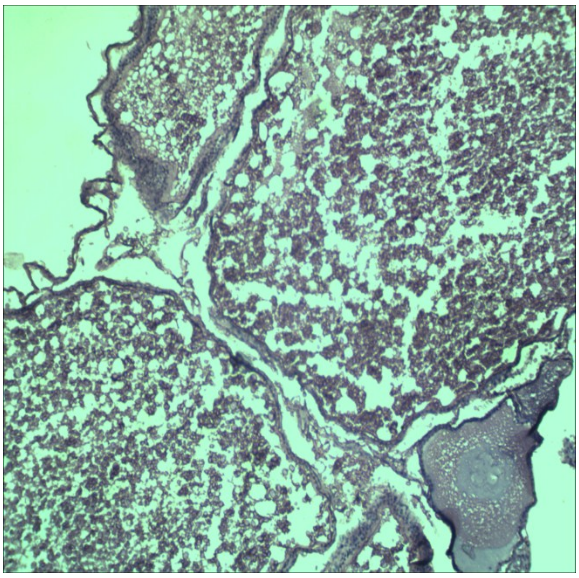

b) Complete necrotic liver of positive control

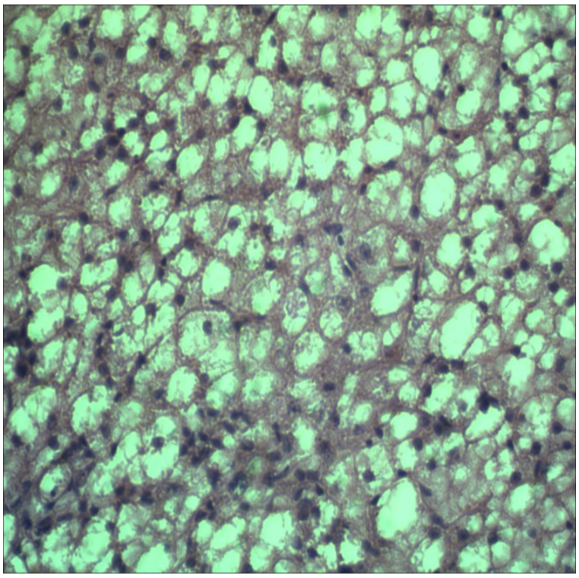

c) Normal testis of positive control

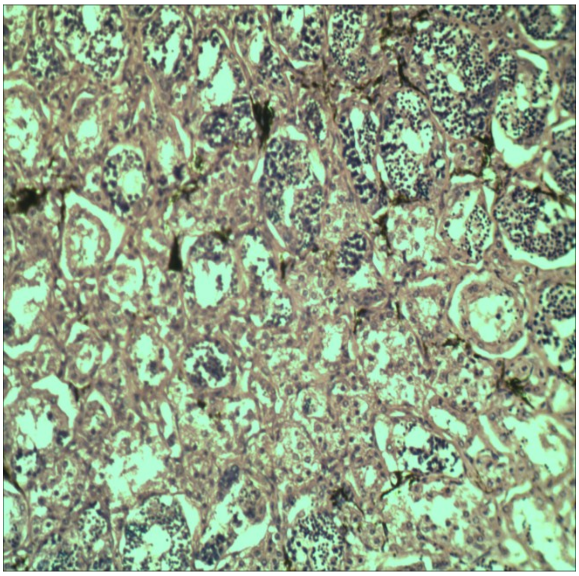

Figure: Sp-6) Histopathology of positive control (a) ovary, (b) liver, (c) testis.

Peaks appeared in case of effluent fingerling. No peaks appeared in control fingerling.

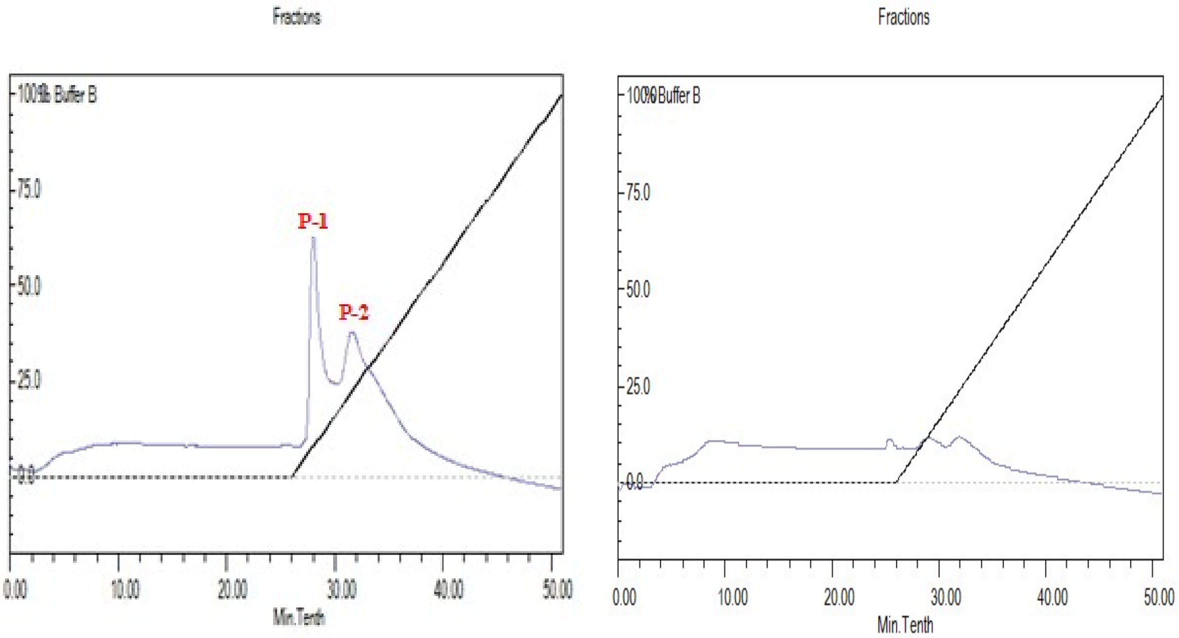

Figure: Sp-7) FPLC of liver fractions showed the vitellogenin peak.

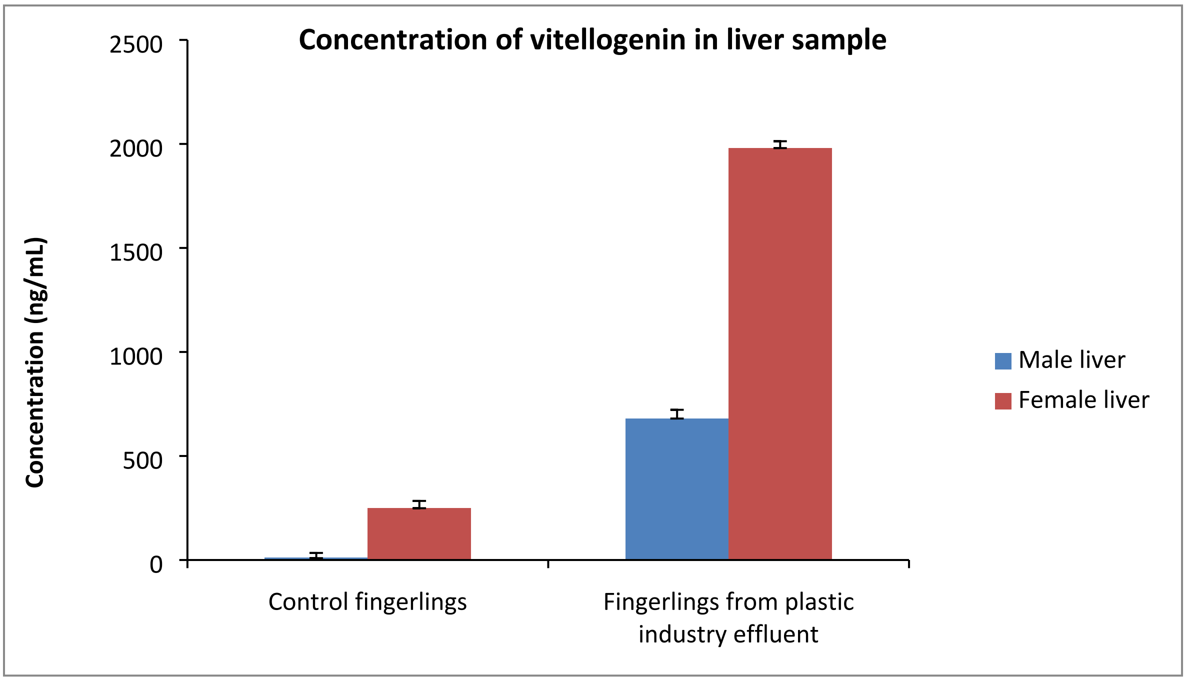

Figure: Sp-8) Quantification of vitellogenin in liver showed higher concentration in the fingerlings collected from plastic industry effluent.

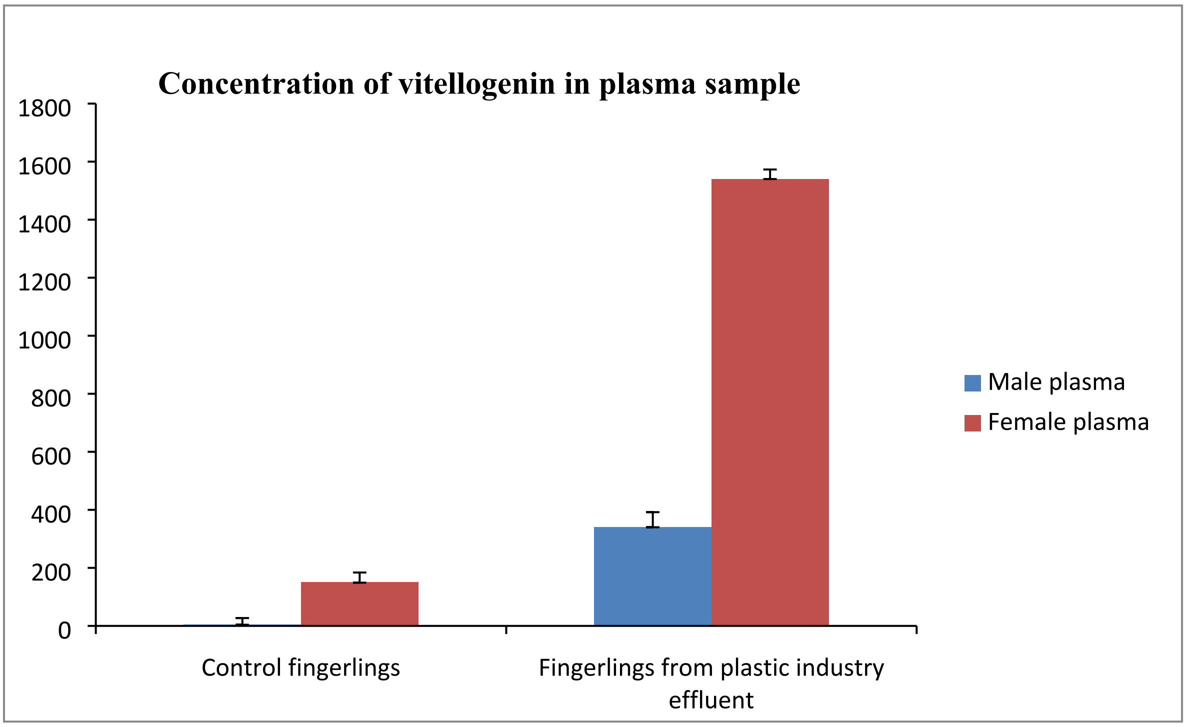

Figure: Sp-9) Quantification of vitellogenin in plasma showed higher concentration in the fingerlings collected from plastic industry effluent.

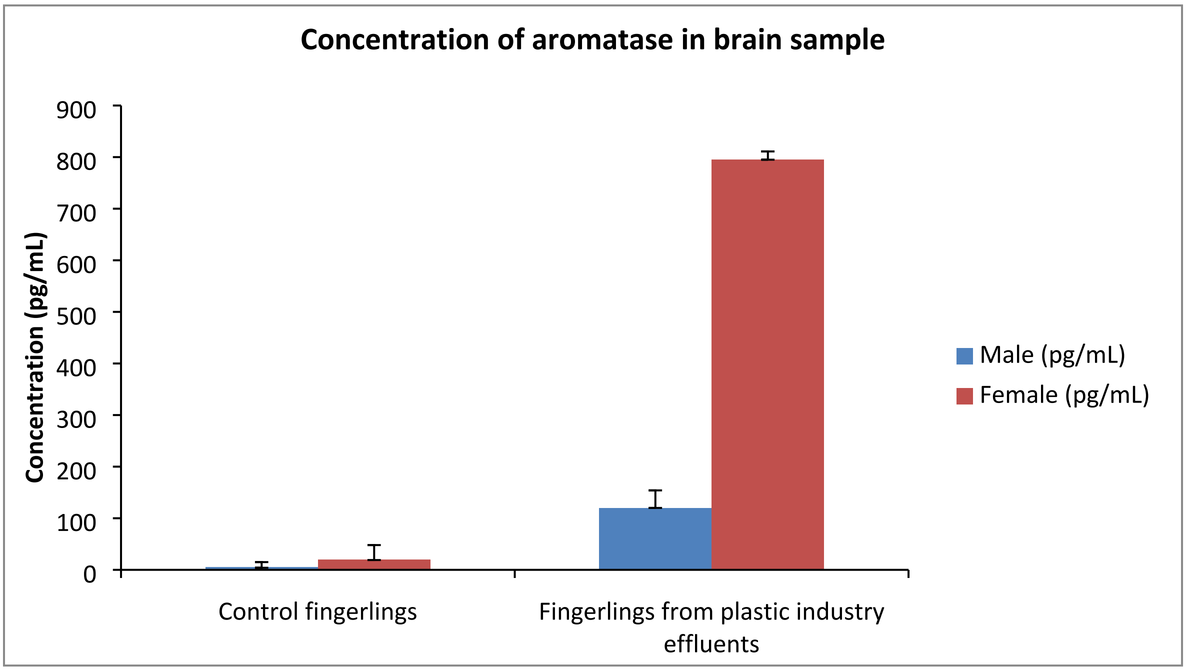

Figure: Sp-10) Quantification of aromatase in brain showed higher concentration in the fingerlings collected from plastic industry effluent.
